# Integrative analysis of multi-omics data reveals inhibition of RB1 signaling promotes apatinib resistance of hepatocellular carcinoma

**DOI:** 10.7150/ijbs.83862

**Published:** 2023-08-21

**Authors:** Ke He, Sanqi An, Fei Liu, Ye Chen, Guoan Xiang, Haihe Wang

**Affiliations:** 1 Minimally Invasive Tumor Therapies Center, Guangdong Second Provincial General Hospital, Guangzhou 510317, China.; 2 Department of General Surgery, Guangdong Second Provincial General Hospital, Guangzhou 510317, China.; 3 Department of Biochemistry, Zhongshan School of Medicine, Sun Yat-sen University, Guangzhou 510080, China.; 4 Department of Critical Care Medicine, Southern Medical University/The First School of Clinical Medicine, Southern Medical University, Guangzhou, 510515, China.

**Keywords:** Apatinib-resistance, Hepatocarcinoma, ATAC-seq, Multi-omics data

## Abstract

Although apatinib is a promising drug for the treatment of liver cancer, the underlying drug resistance mechanism is still unclear. Here, we constructed apatinib-resistant HepG2 cells. We then characterized the epigenomic, transcriptomic, and proteomic landscapes both in apatinib-resistant and non-resistant HepG2 cells. Differential expression, ATAC-seq, and proteomic data analyses were performed. We found that the cell cycle related protein RB1 may play an essential role in the process of apatinib resistant to hepatocarcinoma. Moreover, there were extensive variations at the transcriptome, epigenetic, and proteomic level. Finally, quantitative PCR (qPCR) and western blot analysis showed that expression level of RB1 in apatinib-resistant cell as well as the samples of patients in progressive disease were significantly lower than that in controls. Those results also showed that the RB1 pathway inhibitors CDK2-IN-73 and Palbociclib could relieve the resistance of apatinib resistant cells. Our results further enhance our understanding of the anti-tumorigenic and anti-angiogenic efficacy of apatinib in liver cancer and provide a novel perspective regarding apatinib resistance. Furthermore, we proved that CDKN2B inhibition of RB1 signaling promoted apatinib resistance in hepatocellular carcinoma. Those findings have greatly important biological significance for the resistance of apatinib and the treatment of liver cancer.

## Introduction

Based on the latest statistics, there are 42,030 new cases and 31,780 deaths related to liver cancer every year in the United States 1. Liver cancer incidence has been rising faster than that in any other cancer worldwide 2. Liver cancer is one of the most common cancers, particularly in males for its low overall survival rate 3, 4.

Anti-angiogenesis plays a key role in the treatment of cancer 2, 5-7. Yet, only a few angiogenesis inhibitors are currently clinically available for the treatment of different kinds of cancers. One of these angiogenesis inhibitors, apatinib, has shown satisfactory efficacy in the treatment of breast cancer 5. It has been reported that apatinib is both safe and effective in the treatment of liver cancer 8. Apatinib selectively inhibits the activity of VEGFR2, which negatively affects proliferation, cell migration, and tumor microvascular density mediated by VEGF 8, 9. Clinical trials have shown that apatinib also has an effect on hepatocellular carcinoma (HCC) 10. Since December 31, 2020, the China National Medical Products Administration has officially approved the new indication for the anti-tumor angiogenesis drug of apatinib developed by Hengrui Pharmaceuticals. Apatinib is used for patients with advanced hepatocellular carcinoma who have failed or became intolerable after receiving at least first-line systemic treatment in the past. However, drug resistance to therapies is a major obstacle to successful cancer therapy 11-14. Our knowledge of apatinib resistance in human liver cancer is still insufficient.

Consequently, we attempted to establish apatinib-resistant HepG2 cells and obtained ATAC-seq, RNA-seq, and proteomics data in apatinib-resistant cells using next-generation sequencing. Our study aimed to obtain a global view of the genome, transcriptome, and proteome in apatinib-resistant cells. Finally, we found that the cell cycle and the level of cell cycle-associated proteins, including Retinoblastomal 1 (RB1) are dramatically changed in apatinib-resistant cells. Our results further displayed that inhibition of RB1 signaling promotes apatinib resistance in HCC.

## Methods

### Cell culture and antibodies

HepG2 cells were purchased from American Type Culture Collection (Gaithersburg, MD, USA). All cells were maintained in Roswell Park Memorial Institute 1640 medium (Gibco, Invitrogen, Carlsbad, CA, USA) supplemented with 10% fetal bovine serum (FBS; Gibco/BRL, MD, USA), 100 U/mL penicillin, and 100 mg/mL streptomycin (Beyotime Biotechnology Co., Ltd., Shanghai, China). The cells were cultured at 37°C in 5% CO_2_. Antibodies against anti-rabbitRb1(RB), CDKN2B (p15 INK4B), PCNA, KIAA0101(PAF15), MCM7, and GAPDH were purchased from Cell Signaling Technology, while antibodies againstanti-rabbitCDKN2AIP were purchased from Thermo Fisher Scientific Co., Ltd.

### Screening of apatinib-resistant HepG2 cells

Apatinib-resistant HepG2 cells were screened using a concentration gradient method 15, 16. The initial apatinib (Selleck, Houston, TX, USA) concentration range was 5-10 µM for 48 h followed by normal culture for 24 h, and then a 20% increasing concentration gradient was administered to obtain the first stage drug-resistant concentration cells (HepG2-Apati-P1 cells). Then, HepG2-Apati-P1 cells was administered for 72 h, cultured for 48 h, and then administered according to a 5% increasing concentration gradient to obtain second-stage drug-resistant (HepG2-Apati-P2) cells. After 96 h of administration, 72 h of normal culture, followed by administration of a 1% increasing concentration gradient, the highest concentration of drug-resistant cells was obtained. For IncuCyte analysis, cells were plated as above in 96-well plates, and images were taken with the Real-Time Live-Cell Imaging System (Essen Bioscience, IncuCyte ZOOM, Ann Arbor, MI, USA) 17.

### Identification of apatinib-resistant cells

For cytotoxicity assays, apatinib-treated cells treated with 100 nM of 0.1% dimethylsulfoxide were incubated with 100 nM of YOYO-1 dye (Molecular Probes, Burlington, ON, Canada). We use a Real-Time Live-Cell Imaging System to monitor for more than 72 hours, and regularly take pictures to calculate the degree of cell fusion in different time periods. The degree of cell fusion was used to calculate inhibition rate in each time periods. The 50% growth inhibitory concentrations (IC50s) were calculated using nonlinear regression, fitting to a sigmoidal dose-response curve using a commercially available software program (Prism 6.0, GraphPad Software, San Diego, CA, USA). The drug resistance index (RI) = IC50 of drug-resistant cells/IC50 of parental cells. RI grades 1 to 5 are low resistance, 5 to 15 are moderate resistance, and 15 or more are high resistance 18.

### RNA-seq

Total RNA was extracted from cells using TRIzol reagent (Invitrogen) according to the manufacturer's instructions. First, the extracted total RNA was removed with a biotin-labeled specific probe (Ribo-Zero ™ rRNA Removal Kit) to remove ribosomal rRNA. After purification, the RNA was fragmented under a certain temperature and ionic environment. Then, random primers and reverse transcriptase were used in the TruSeq® Stranded kit to synthesize the first strand of cDNA, followed by the use of DNA polymerase I and RNaseH to synthesize the double-stranded cDNA. During second-strand cDNA synthesis, the RNA template was removed and dTTP was replaced by dUTP, which prevents the second strand of cDNA from being amplified in the subsequent process, for the reason that the polymerase could not cross the dUTP site on the template during extension 19. The double-stranded cDNA product was subsequently ligated with an "A" base and a linker. The ligation product was amplified and the final cDNA library was obtained after purification. Finally, the constructed sequencing library was sequenced pair end on the BGISEQ-500/ MGISEQ-2000 System (BGI-Shenzhen, China).

### ATAC-seq

Cells were harvested and then intact and homogenous HepG2-Apati cells were counted, followed by centrifugation of 50,000 cells for 5 min at 500 g at 4°C. The cells were then washed once with 50 μl of cold phosphate-buffered saline (PBS) buffer and centrifuged again under the same conditions after removing and discarding the supernatant. After removing and discarding supernatant again, the pellet was gently resuspended in 50 μl of cold lysis buffer followed by centrifugation for 10 min 20. After discarding the supernatant, resuspension of the nuclei pellet was carried out immediately using the prepared transposition reaction mix which contained Nextera Tn5 Transposase from Nextera kit. The transposition reaction was incubated at 37°C for 30 min. A Qiagen MinElute PCR Purification Kit was used followed by the application of 10 μl elution buffer (Buffer EB from the MinElute kit consisting of 10 mM Tris·Cl, pH 8) to elute transposed DNA. Finally, PCR amplification was performed to amplify transposed DNA fragments. Prior to amplification, adapters have to be completed with a 72°C extension step, as well as the qPCR and library quality control using gel electrophoresis is provided. Libraries were pooled at equimolar ratios with barcodes and sequenced on the BGISEQ-500 platform (BGI-Shenzhen, China) 21.

### Proteomics

HepG2-Apati and HepG2 cells were cultured on NHA for 24 h. Cells cultured in a Petri dish were used as a control. Cell lysis was performed to extract total proteins. The proteomics analysis was performed by Genome Technology Co. Ltd. (Beijing, China). The prepared samples were labeled with 8-plex iTRAQTM (Applied Biosystems, Foster City, CA, USA) and analyzed using 2D strong cationic exchange/reversed phase liquid chromatography matrix-assisted laser desorption/ionizationtandem mass spectrometry (MDS Sciex 4800 PLUS, Applied Biosystems Inc., SCX/RP-HPLC-ESI-MS/MS) 22. The iTRAQ data were searched with ProteinPilot software v3.0 (Applied Biosystems/MDS-Sciex) for protein identification and quantification. The experiment was performed in two independent runs.

### Processing of the ATAC-seq and RNA-seq data

Raw data of ATAC-seq and RNA-seq have been deposited in https://www.ncbi.nlm.nih.gov/sra (accession no. PRJNA678418). The reads of ATAC-seq were mapped to the hg19 human genome using bowtie2^23^. We used Macs2 (v1.3.4d) to calculate the peaks of Ensembl annotated genes. ATAC-seq peaks were identified according to the Macs2 methods 24. Then, the annotation of ATAC-seq peaks was performed using “ChIPseeker” and "TxDb.Hsapiens.UCSC.hg19.knownGene" packages implemented in R. The differential analysis of ATAC-seq peaks was carried out using the “DiffBind” package implemented in R. Short reads of RNA-seq were mapped to the hg19 human genome using hisat2^25^. We used cufflinks (v1.3.4d) to calculate the FPKM of Ensembl annotated genes. Gene Ontology (GO) analysis was achieved through DAVID (https://david.ncifcrf.gov/) with the genes expressed (TPM >1) as the background 26. We used differently expressed genes as input data of DAVID database, and select the “Homo sapiens” species. Gene annotation enrichment analysis was performed. R was used to plot the result of gene annotation enrichment analysis from DAVID.

The data were visualized using the Integrative Genomics Viewer (IGV) tool 27. Differential gene expression analyses were performed based on the input data using DESeq2^28^ according to the read counts of each gene determined by HTSeq 29. The genes with false discovery rate (FDR) < 0.05 and mean counts per million (CPM) > 100 were determined to be differentially expressed genes. Then, we use a Wilcoxon test statistic on the log fold changes of ATAC-seq signal (Apatinib/NC). This test will reject the null hypothesis which suggests that no statistical relationship and significance exists. The log fold change is chosen here would be sensitive to differences.

### Processing of proteome data

For the quantitative proteome, the 'ProteinGroups' output files generated by the MaxQuant search were input into Perseus software. Proteome-wide label-free quantification was executed in MaxQuant^30^, ^31^. Subsequent data analysis and visualization were completed using Perseus 32. GO analysis was also performed using DAVID with the genes as we described before. Finally, A binary (yes /no) interaction matrix of differentially expressed proteins obtained from the search tool for retrieval of interacting genes (STRING) (https://string-db.org) database to show the interaction map of these proteins 33. STRING integrates both predicted and known protein-protein interaction, can be used to predict functional interactions of proteins. To seek potential interactions between differentially expressed proteins, the STRING tool was employed. Active interaction sources, including experiments, databases, text mining, and co-expression. In the networks, the nodes correspond to the proteins and the edges represent the interactions.

### Tissue specimens and Immunohistochemical staining

This research was conducted under the approval and supervision of the Ethics Committee of Guangdong Second Provincial General Hospital. Five recruited HCC patients without any preoperative chemotherapy or radiation therapy had signed the written informed consent prior to the study. Through percutaneous liver biopsy, we obtained liver metastasis samples during partial response (PR) and progressive disease (PD) periods following oral administration of apatinib (Hengrui Pharmaceutical Co., Ltd., Jiangsu, China) in the above five patients. HCC tissues were flash frozen in liquid nitrogen and stored at -80°C until RNA extraction 34.

Before used, all cases were diagnosed by two certificated pathologists without discrepancy. The paraffin-embedded tissues were first stained with hematoxylin and eosin (HE) for histological examination. Subsequently, deparaffinized sections were treated with 3% H2O2 and subjected to antigen retrieval by citric acid (pH 6.0). After overnight incubation with primary antibody (anti - RB1 and CDKN2B antibody; Boster Bio, Pleasanton, USA) by 1:200 at 4 °C, sections were incubated for 15 minutes at room temperature with horseradish peroxidase-labeled polymer conjugated with secondary antibody (MaxVision Kits) and incubated for 1 minute with diaminobenzidine. The sections were then lightly counterstained with hematoxylin 35. The sections without primary antibody were served as negative controls. Expression level of RB1 and CDKN2B was determined according to the average score of two pathologists' evaluations based on the intensity and extent of staining. The staining intensity was graded as follows (intensity score): 0, negative staining; 1, weak staining; 2, medium staining; 3, strong staining. The following method was used to determine the proportion of stained cells in each specimen (proportional score): 0, <1%; 1,1% -25%; 2, 26% -50%; 3, 51% -75%; 4, > 75%. The histological score (H-score) of each specimen was calculated using the following formula: H-score = proportional score × intensity score. Samples with H-score > 4 were considered as strong positive, H-score ≤ 4 was weak positive, and H-score ≤ 1 was negative 36.

### Quantitative real-time PCR

Using the RNA isolation kit (Takara Biomedical Technology [Beijing] Co., Ltd.) to extract total RNA from cell lines, we used MMLV reverse transcriptase to synthesize cDNA using the SYBR Green reaction system (Takara Biomedical Technology [Beijing] Co., Ltd.) to amplify cDNA 37. GAPDH was used as an internal reference to analyze the differences in mRNA expression levels of RB1, CDKN2B, CDKN2AIP, PCNA, KIAA0101, and MCM7. The PCR sequences were as follows: RB1: sense: 5'-GGAAGCAACCCTCCTAAACC-3', antisense: 5'-TTTCTGCTTTTGCATTCGTG-3'; CDKN2B: sense: 5'-CCAGAAGCAATCCAGGCGCG-3', antisense: 5'-CGTTGGCAGCClTCATCG-3'; CDKN2AIP sense: 5′- ATGGGCCAACAACGTGTTTC-3′, antisense: 5′-GAAAACAGGGGCATCCTCCA-3′; PCNA: sense: 5′-CCATCCTCAAGAAGGTGTTGG-3′, antisense: 5′-GTGTCCCATATCCGCAATTTTAT-3′; KIAA0101: sense: 5′-CCCAGGCAACATAGCGTAAA-3′, antisense: 5′-GGATGGTCTCGATCTCCTGA-3′; MCM7: sense: 5′-TCAATTTGTGAGAATGCCAGGCGC-3′, antisense: 5′-CACAGTTACCAACTTCCCCACAGA-3′; GAPDH: sense: 5′-TCCTGCACCACCAACTGCTTAG-3′, antisense: 5'-TCTTACTCCTTGGAGGCCATGT-3'.

### Western blot

After trypsin digestion of cells and tissue samples, RIPA protein lysis solution and protease inhibitors were added, and samples were placed on ice for 20 min and centrifuged at 4°C for 10 min at 12,000 rpm. The supernatant was transferred to a microfuge tube and stored at -80℃ for later use. The BSA protein standard (0.5 mg/ml) and DEPC water were diluted to create five protein standard solutions of different concentrations. Then, 200 μL BCA working solution was added and samples were incubated in a 96-well plate at 37°C. The absorbance was measured at 562 nm, a standard curve was prepared, and the protein concentration of each sample was measured.

### Cell proliferation

The Cell Counting Kit-8 (CCK8) was used to detect proliferation. Cells in logarithmic growth phase were tryps inized at a density of 5 × 10^4^/mL in a 96-well plate and incubated for 24 h followed by the addition of various concentrations of apatinib, CDK2-IN-73 (Selleck, Houston, TX, USA), Palbociclib (Selleck), or 10% FBS to each well. After 72 h, 10 μL of CCK-8 reagent was added to each well, and the samples were incubated at 37°C for 1 h. The absorbance was measured at 450 nm, and each experiment was performed at least three times.

### Statistical analysis

All wet-lab experiments were repeated three times, and the results are expressed as mean ± standard deviation. Student's t-test was used to analyze statistical differences between apatinib group and control group for wet-lab experiments. The graph was made using GraphPad Prism 6 (La Jolla, San Diego, CA, USA), and SPSS V17 Student Edition Software was used for statistical analysis. P < 0.05 was considered statistically significant for wet-lab experiments.

## Results

### Establishment of apatinib-resistant HepG2 cells and their general biological characteristics

**Figure 1:**
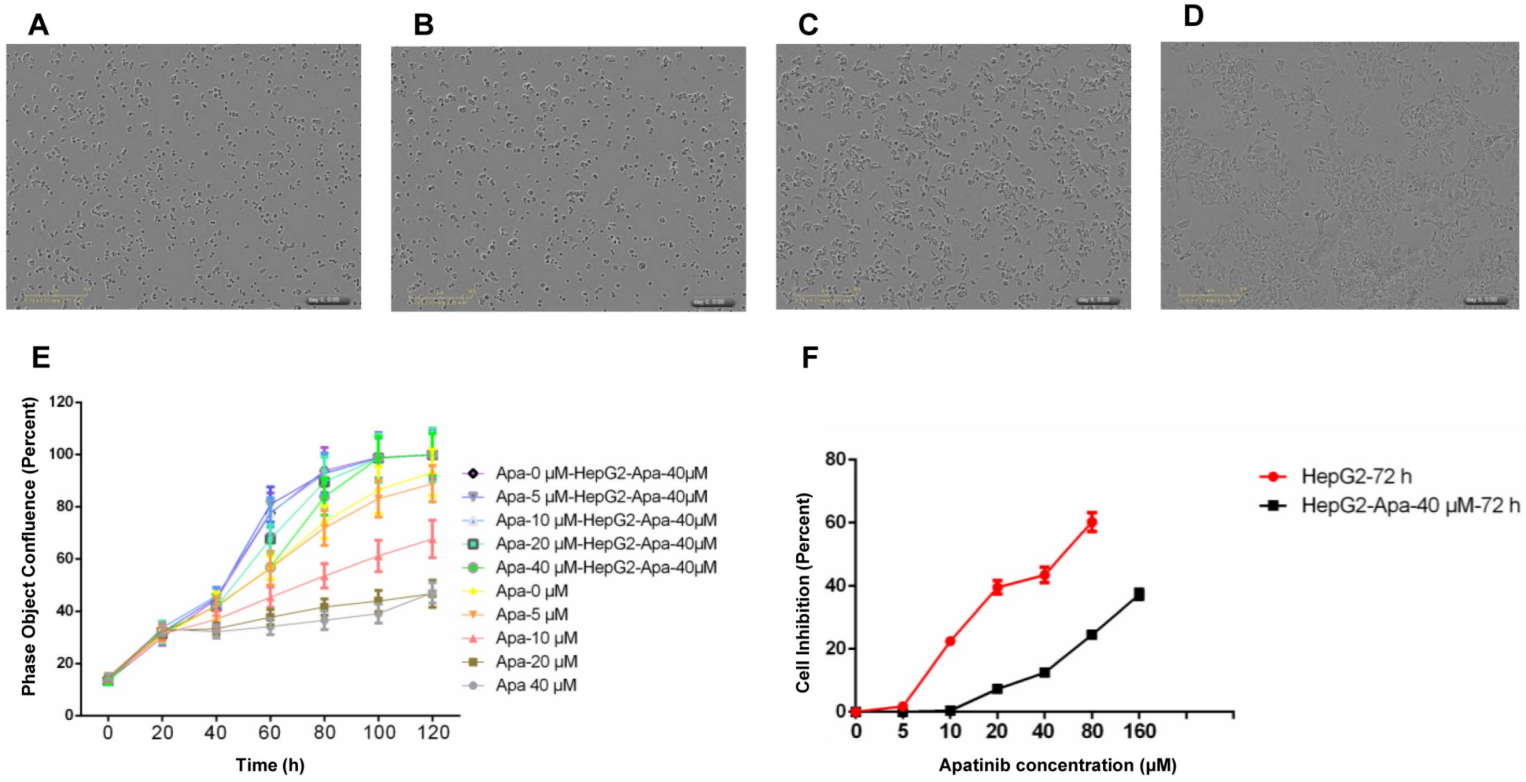
** Construction and identification of HepG2-resistant cells.** A. HepG2 cells. B. HepG2-resistant cells resistant to apatinib, with a drug resistance concentration of 40 μmol/ml. C. HepG2 cells treated with apatinib at 40 μmol/ml for 120 h. D. HepG2-apatinib-40μM cells treated with 40 μmol/ml apatinib for 120 h. E. Proliferation curves (real-time dynamic cell fusion) of HepG2 and HepG2-apatinib-40μM cells after treatment with different concentrations of apatinib. F. Proliferation inhibition curves of apatinib for HepG2 and HepG2-apatinib-40μM cells.

We used intermittent, repetitive, and increasing concentration gradient methods to establish apatinib-resistant HepG2 cells, with a drug resistance concentration of 40 μmol/ml. Compared with parental cells, HepG2-apatinib-40 μM cells exhibit overlap, aggregation, growing volume, cytoplasmic vacuole formation, increasing granules, and the formation of scattered giant cells. The number of chromosomes increased, hyperdiploidy was evident, and the division index was low (Fig. 1A, B). These cells can proliferate at an apatinib concentration of 40 μmol/ml (Fig. 1C, D). The *in vitro* cell growth and proliferation curve showed that HepG2-apatinib-40 μM cells reached a peak in 4 to 5 days, while the growth and proliferation of HepG2 cells at the same concentration significantly declined (Fig. 1E). The effect of the concentration of apatinib on the proliferation of HepG2-apatinib-40μM resistant strain cells was much greater than that of HepG2 cells (Fig. 1F). The linear regression method determined that the IC50 values of apatinib for HepG2-apatinib-40 μM and HepG2 cells were 268.8 μmol/ml and 43.66 μmol/ml, respectively. The resistance index of HepG2-apatinib-40μM cells to apatinib was 6.15, which demonstrated moderate resistance.

### Changes in chromatin accessibility of apatinib-resistant cells

**Figure 2:**
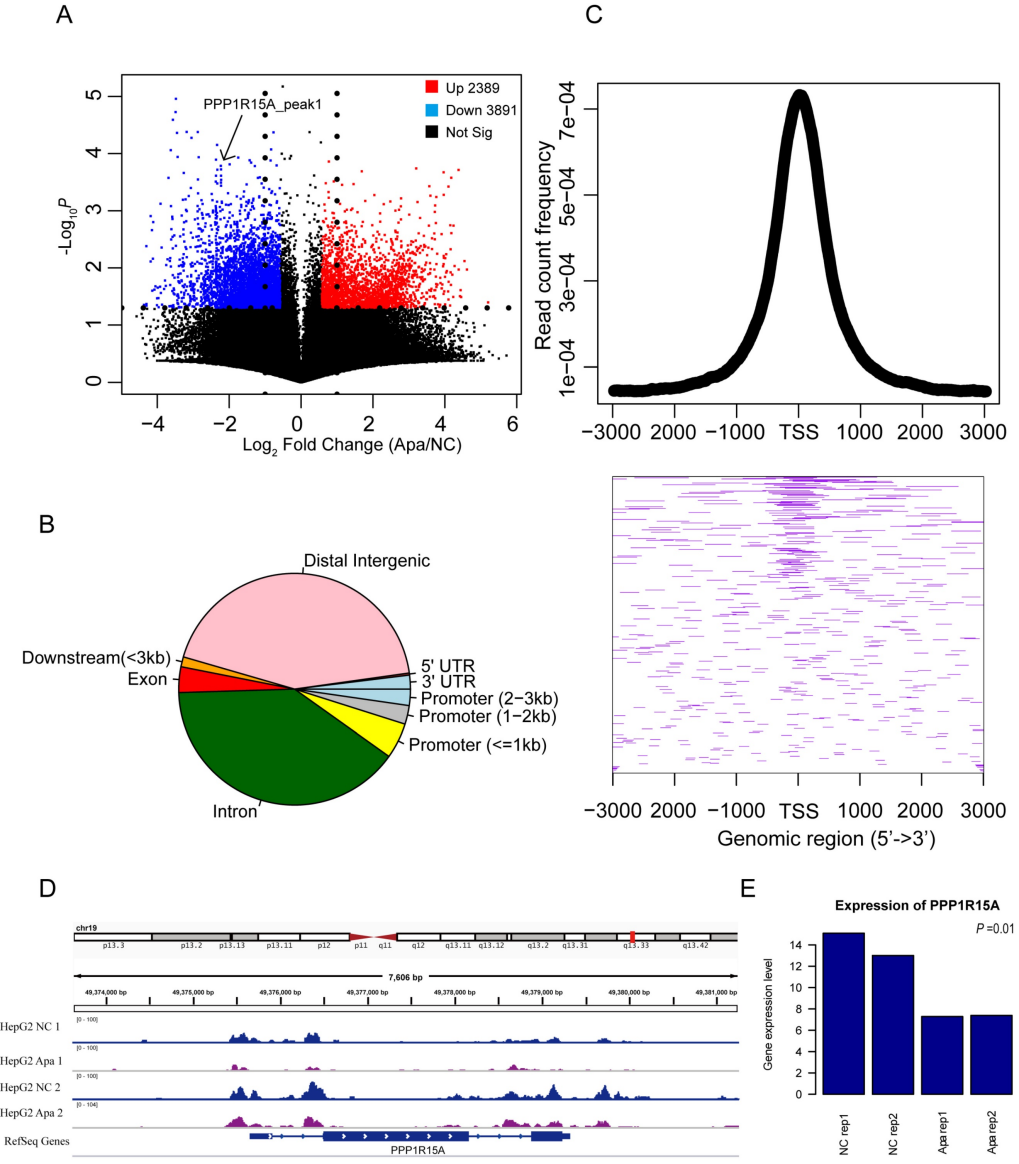
** Changes in chromatin accessibility of resistant HepG2 cells.** A. Volcano plot showing the differentially ATAC-seq peaks between apatinib-resistant cells and untreated HepG2 cells, there are 3,891 peaks were increased in chromatin accessibility and 2,389 peaks were decreased based on read counts in apatinib-resistant cells. B. Pie chart of the genomic location distribution of different levels of ATAC-seq sites. C. The average profile of the ATAC-seq signals is a graph showing the read count frequency in the range from -3000 bp to +3000 bp (Up); Heatmap of different ATAC-seq signals in region around TSS (Down). D. Genome tracks showing a comparison of apatinib-resistant HepG2 ATAC-seq profiles to untreated HepG2 ATAC-seq profiles in PPP1R15A. E. The mRNA expression levels of PPP1R15A in apatinib-resistant versus untreated HepG2 cells.

It is unknown if chromatin accessibility is related to drug resistance. The ATAC-Seq profiles in apatinib-resistant cells compared with HepG2 cells is shown in Figure 2A. Apatinib-resistant cells were likely to have less accessible chromatin regions than HepG2 cells, because the down-regulated peaks were more than the up-regulated peaks in Figure 2A, and these different accessible chromosomes were clustered in transcription start site (TSS) regions (Fig. 2B, C). An example of ATAC-seq read counts in the TSS region of Protein Phosphatase 1 Regulatory Subunit 15A (PPP1R15A, also known as GADD34) was presented (Fig.2D). Theoretically, the expression level of genes was different between apatinib-resistant and HepG2 cells. Further, we found that there was significant discrepancy in gene expression among several essential genes including PPP1R15A (Fig. 2E). PPP1R15A has an effect on rates of translation initiation and thereby protein synthesis in response to stressful conditions 38. It gives us a hint that proteomic changes in progression of apatinib-resistance. By analyzing gene sequences enriched in ATAC-seq differential sites between drug-resistant strains and wild-type cells, we found that these differential sites were mainly enriched in CTCF, BORIS, Jun-ap1, and Fra1 proteins, indicating that these differential sites would be affected by transcription factors. Universal regulation plays an important role in regulating gene expression (Supplemental Figure 1).

### Transcriptome sequencing of apatinib-resistant cells

**Figure 3:**
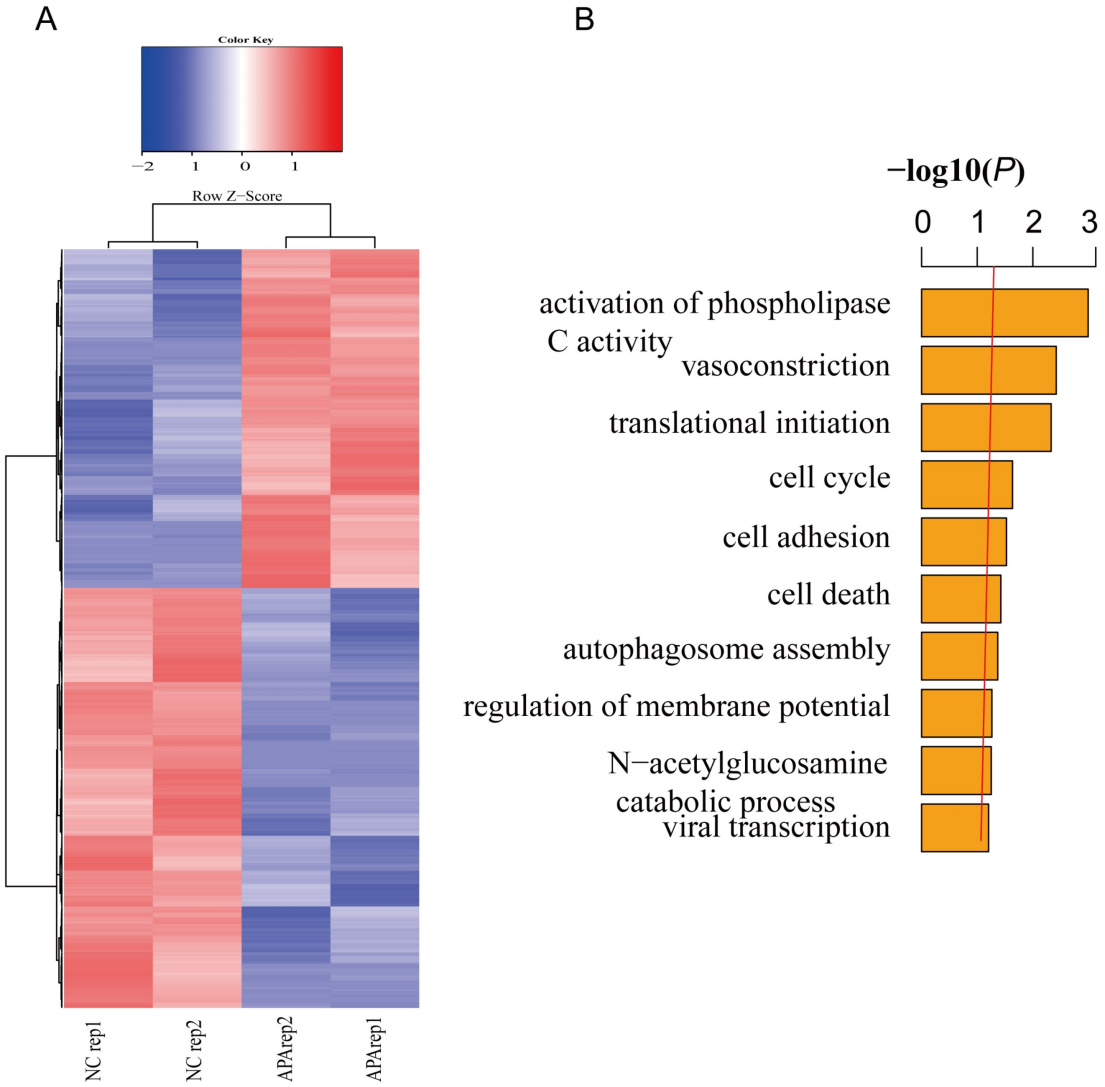
** Transcription profiles in resistant and non-resistant HepG2 cells.** A. Heatmap shows significantly (FDR ≤0.05) differentially expressed genes across two comparisons (blue, low expression; red, high expression). B. GO analysis showed that differentially expressed genes are involved in the activation of phospholipase C activity, vasoconstriction, translational initiation, cell cycle, and so on.

To further verify the relationship between global gene expression level and patterns of chromatin accessibility in apatinib-resistant cells and untreated HepG2 cells, we performed gene differential expression analysis to obtain a list of statistically differential genes (i.e., all genes with FDR < 0.05) in apatinib-resistant and non-resistant HepG2 cells (Fig. 3A). Then, we identified GO terms that are statistically enriched in this gene list. Gene expression level analysis showed that a range of differentially expressed genes were down-regulated in apatinib-resistant cells and the most significant GO terms were phospholipase C activity, vasoconstriction, translational initiation, and cell cycle (Fig. 3B, Supplemental Table-1). We concluded those biological functions were highly related to the apatinib resistance, especially for vasoconstriction, translational initiation, and cell cycle. Apatinib is a tyrosine kinase inhibitor that suppresses vascular endothelial growth factor receptor-2 (VEGFR2). Thus, it is easy to understand that the GO terms are enriched in vasoconstriction 39, 40. How translational initiation and the cell cycle affect apatinib resistance is less clear.

### Changes in chromatin accessibility are consistent with global transcriptome changes

**Figure 4:**
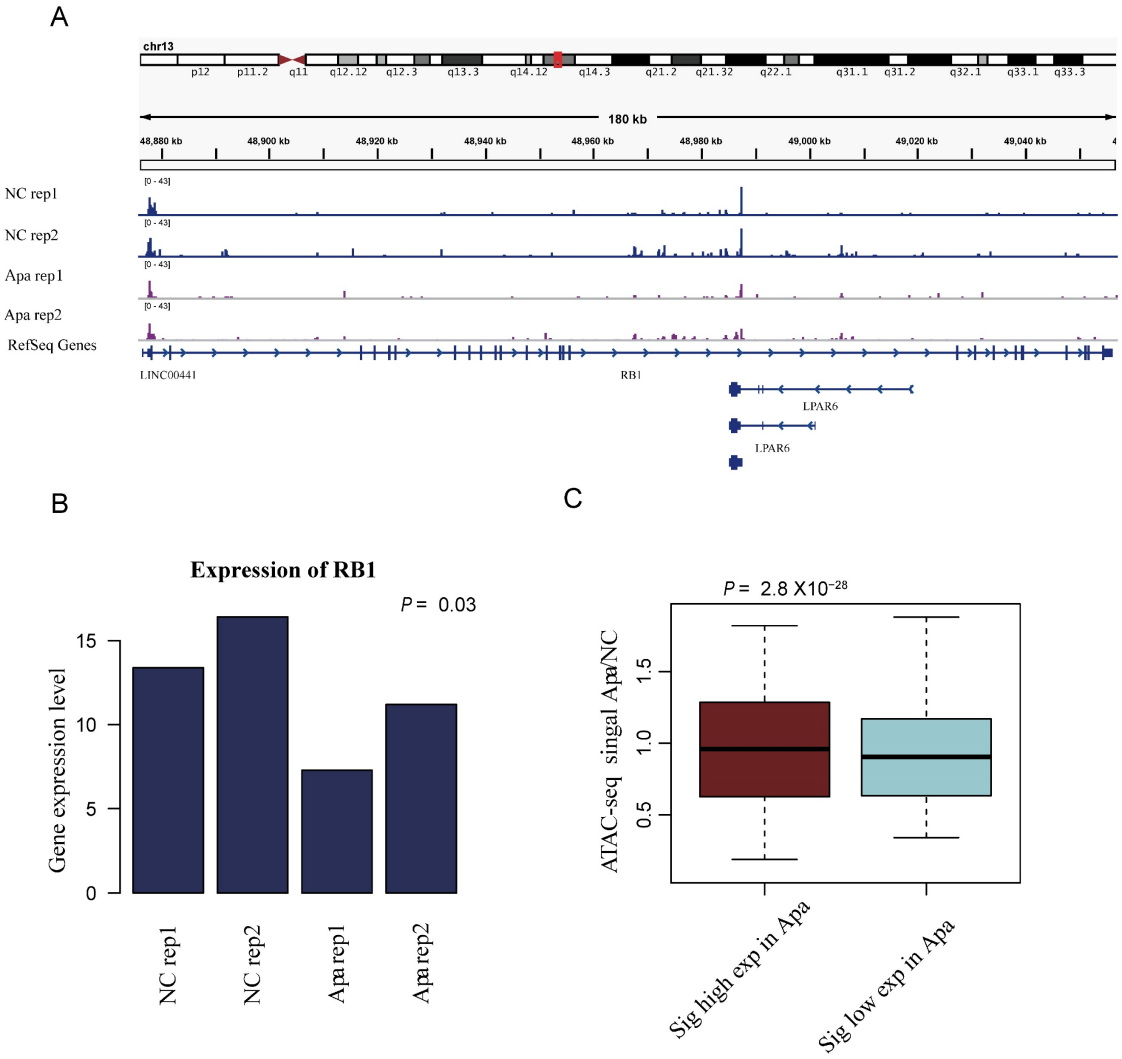
** Changes in chromatin accessibility are consistent with global transcriptome changes.** A. Genome tracks showing a comparison of ATAC-seq profiles of RB1 in apatinib-resistant and non-resistant HepG2 cells. B. The mRNA expression levels of RB1 in apatinib-resistant versus untreated HepG2 cells, statistical analysis was performed by Student t-test. C. Boxplots of chromatin accessibility values for high expression genes and low expression genes in apatinib-resistant cells.

Our analysis indicated that the changes in chromatin accessibility are consistent with global transcriptome changes. To further understand the relationship between translational initiation and drug resistance of apatinib, as exemplified in Figure 4A, the ATAC-seq peak for the Retinoblastoma-associated protein RB1 was down-regulated in apatinib-resistant cells. The expression level of RB1 was also down-regulated in apatinib-resistant cells (Fig. 4B). Eukaryotic translation initiation factor 2A (EIF2A), which catalyzes the formation of puromycin-sensitive 80S preinitiation complexes, was down-regulated in apatinib-resistant cells (data not shown). The highly expressed genes in apatinib-resistant cells had a higher ATAC-seq signals, while the genes with lower expression had lower ATAC-seq signals (Fig. 4C). Overall, the results showed that the whole region of open chromosome status was significantly correlated with the gene expression level.

### Proteome profiles support the result that the cell cycle plays a key role in apatinib-resistance

**Figure 5:**
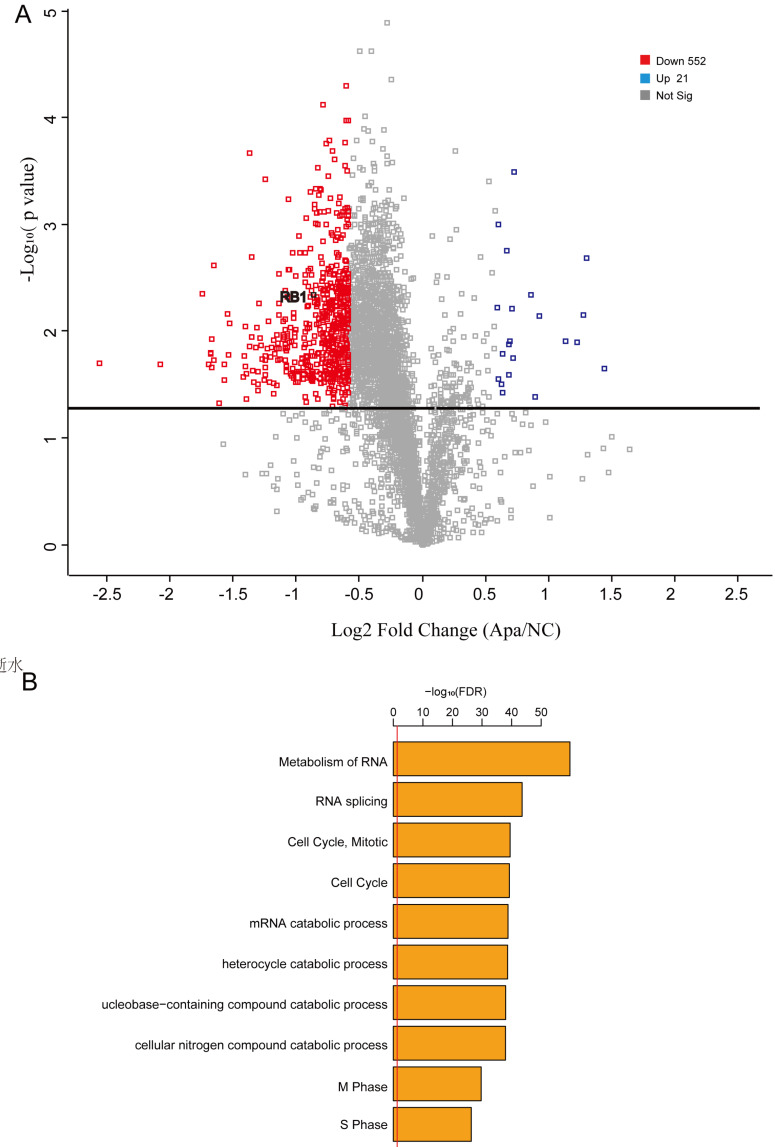
** Proteome profiles in apatinib-resistant cells support the result that the cell cycle plays a key role in apatinib resistance.** A. Volcano plot reflecting the results from the statistical analysis of the proteins quantified among apatinib-resistant and non-resistant HepG2 cells, statistical significance was considered for FDR < 0.05, statistical analysis was performed by Student t-test. B. GO analysis showed that a number of differentially expressed proteins are involved in the cell-cell adhesion, regulation of mRNA stability and cell cycle.

Integrating proteomics data is more accurate than integrating transcriptomics for most conditions. Thus, we performed quantitative proteomics to analyze proteome profiles in apatinib-resistant and untreated HepG2 cells. We used Proteome Profilers to elucidate the protein profiles in apatinib-resistant and non-resistant cells. We generated a volcano plot to illustrates significantly differentially abundant proteins in apatinib-resistant cells compared with HepG2 cells, which showed that the level of Rb1, a well-known tumor suppressor gene, decreased significantly in apatinib-resistant cells (Fig. 5A). Apatinib-resistant cells enriched in some important pathways including cell cycle, metabolism of RNA, and RNA splicing (Fig. 5B, Table. s1). A total of 45 genes are differentially expressed at both mRNA and protein levels (Supplemental Table-2). Bioinformatics STRING (Protein-Protein Interaction Networks Functional Enrichment Analysis) analysis of the proteomic data from the label-free mass spectrometric study of different expressed proteins in apatinib-resistant and untreated HepG2 cells was performed (Supplemental Figure 1).

### Expression of RB1 pathway targets at the cellular level and in clinical samples

**Figure 6:**
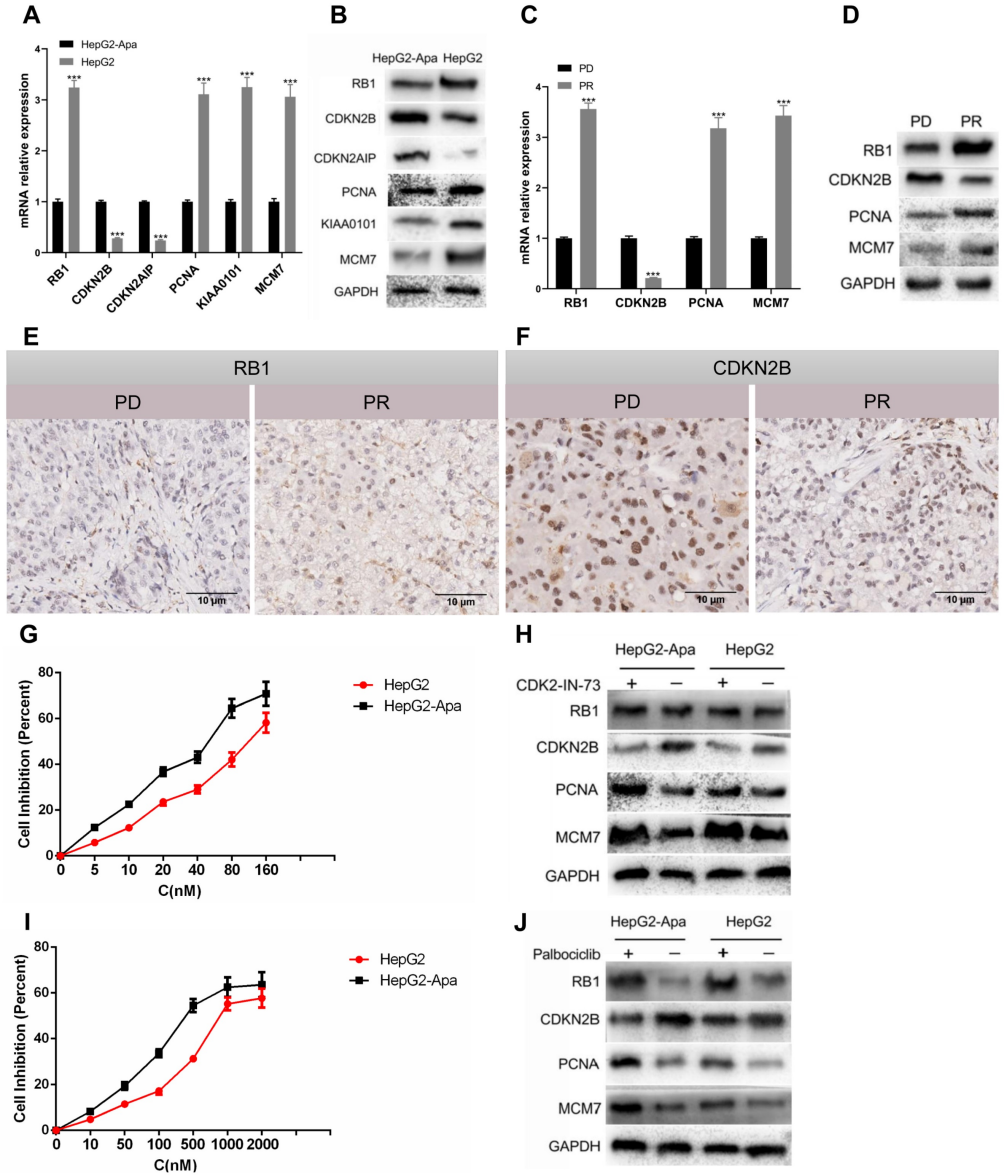
** Expression status of RB1 pathway targets in HCC cells and in clinical samples; CDK2-IN-73 and Palbociclib can relieve apatinib resistance.** A. qPCR analysis showed that the expression levels of RB1, PCNA, KIAA0101 and MCM7 in HepG2-resistant cells were significantly reduced, and the expression levels of CDKN2B andCDKN2AIP were increased. B. Western blot detection of the above proteins is consistent with qPCR detection results. C. Analysis of mRNA expression of five PD patients showed high expression of CDKN2B and low expression of RB1, PCNA, and MCM7. D. Western blot analysis showed that PD patients had high expression of CDKN2B but low expression of RB1, PCNA, and MCM7. E. Representative photographs of staining of RB1 protein in a pair of HCC patients tissues in PD and PR (20X). F. Representative photographs of staining of CDKN2B protein in a pair of HCC patients tissues in PD and PR (20X). G. The proliferation inhibition curves of resistant HepG2 cells and HepG2 cells treated with CDK2-IN-73. H. Western blot analysis showed that the expression of CDKN2B in HepG2resistant cells was not significantly different, and the expression levels of RB1, PCNA and MCM7 were upregulated. I. Proliferation inhibition curves of HepG2 resistant cells and HepG2 cells treated with Palbociclib. J. Western blot analysis results showed that the expression levels of RB1, PCNA, and MCM7 were upregulated. The bars represent mean ± standard deviation; ∗*P* < 0.05.

Subsequently, we used qPCR to detect the mRNA expression levels of RB1, CDKN2B, CDKN2AIP, PCNA, KIAA0101, and MCM7 in resistant and non-resistant HepG2 cells. The expression levels in the cells were significantly reduced, and the expression levels of CDKN2B and CDKN2AIP increased (Fig. 6A), consistent with multi-omics analysis. Western blotting was used to detect the expression level of the proteins encoded by the above genes, and the results were consistent with the qPCR detection trend (Fig. 6B).

We biopsied tumor specimens from five patients with PR and PD to detect the protein and mRNA levels of RB1, CDKN2B, PCNA, and MCM7. qPCR revealed that CDKN2B was highly expressed in PD patients, while RB1, PCNA, and MCM7 exhibited low expression (Fig. 6C), which was consistent with the expression levels in HepG2 resistant cells, indicating that the development of drug resistance in clinical liver cancer patients is closely related with RB1 Signaling pathway (Fig. 6D). We also examined the expression levels of RB1 and CDKN2B in tumor specimens from five patients with PR and PD using immunohistochemical staining. RB1 and CDKN2B were expressed in nucleus, which was consistent with previous reports. Our results indicated that RB1 exhibited low expression (Fig. 6E), and CDKN2B was highly expressed in PD patients (Fig. 6F). The results of PD samples detection at the protein level were consistent with mRNA expression.

### Inhibitors CDK2-IN-73 and Palbociclib can relieve the resistance of cells to apatinib

The CDK inhibitor, CDK2-IN-73 was used to treat HepG2 resistant and non-resistant HepG2 cells, and CCK8 was used to detect cell viability and drug resistance. The two groups of cells were treated with different concentrations of CDK2-IN-73 for 72 h. The IC50 of HepG2 resistant cells was 41.8 μM, while the IC50 of HepG2 cells was 120 μM (Fig. 6G), indicating that CDK2-IN-73 has a lower IC50 in drug-resistant cells. Drug-resistant cells were resistant to apatinib, while the low expression of CDKN2B in HepG2 non-resistant cells was not sensitive to the inhibitory effect of CDK2-IN-73. Changes in the expression of RB1, CDKN2B, PCNA, and MCM7 were detected in the two groups of cells. In resistant cells, RB1, PCNA, and MCM7 were highly expressed while CDKN2B expression was low (Fig. 6H).

Palbociclib is an inhibitor of CDK4/6, which is an important target of the RB1 pathway. Palbociclib was used to treat HepG2 resistant and non-resistant cells. CCK8 is used to detect cell viability and whether drug resistance is released. The two groups of cells were treated with different concentrations of Palbociclib for 72 h. The IC50 of HepG2 resistant cells was 0.42 μM while the IC50 of non-resistant cells was 0.89 μM (Fig. 6I), indicating that Palbociclib can relieve apatinib resistance. The expression levels of RB1, CDKN2B, PCNA, and MCM7 were detected in the two groups of cells. RB1, CDKN2B, PCNA, and MCM7 were highly expressed in HepG2 resistant cells, indicating that CDK4/6 was inhibited, Resulting in RB1 activation and subsequent induction of apoptosis (Fig. 6J). Therefore, Palbociclib was a candidate therapeutic target for the treatment of apatinib resistance.

## Discussion

In 2020, the Phase III registered clinical study of apatinib for second line and above treatment of advanced HCC (the AHELP study) was released at the ASCO annual meeting oral report: the study included a total of 393 patients who had previously received at least first-line systemic treatment patients with advanced HCC who failed or could not tolerate later. The results showed that apatinib could significantly prolong the median overall survival of patients: 8.7 months in the apatinib group versus 6.8 months in the placebo group. The median progression-free survival of the apatinib group was 4.5 months, which was also significantly higher than the 1.9 months of the control group. Based on the exciting results of this study, in second-line treatment of advanced HCC, the new guidelines officially include apatinib as a treatment recommendation; the recommendation level is level I, and the level of evidence is category 1A. However, we see that the effect of apatinib in the treatment of liver cancer is not satisfactory, which might be closely related to its drug resistance.

In this study, RB1 was dramatically changed in apatinib-resistant cells contrast to normal cells. Moreover, the mRNA expression levels of five PR and PD biopsy tumor samples were detected, and it was found that RB1 and downstream genes of PD biopsy tumor samples showed low expression. Additionally, we characterized the epigenomic, transcriptomic, and proteomic landscape in apatinib-resistant cells in HCC. This conclusion will further accelerate our understanding of the anti-tumorigenic and anti-angiogenic efficacy of apatinib in liver cancer and provide a novel perspective regarding apatinib resistance.

Omic-technologies have a broad range of applications. Soon, large-scale omic data in specific biological systems will be available. We consider it is a great advantage to apply our computational strategies to these systems to uncover new mechanisms that may be important for specific systems. For instance, applying the framework to a population of cancer samples may reveal novel cancer target involved in tumorigenesis in certain types of cancers.

Drug resistance is a well-known phenomenon that occurs when cancer is resistant to drug treatment. Some drug-resistant methods are disease-specific, while others, such as drug efflux observed in microorganisms and human drug-resistant tumors, are evolutionarily conserved 41-45. Although many types of cancer are initially susceptible to chemotherapy 41-43, 46-50, they can subsequently develop resistance through certain underlying mechanisms, such as DNA mutations, cell cycle, stem cell and metabolic changes that promote drug inhibition and degradation 51-61.

RB1 is a well-known cancer suppressor gene 62, 63. It has been reported that Rb1 and its metabolite compound K can effectively suppress cancer stem cell self-renewal without regrowth, and Rb1 and compound K treatment also sensitized cancer stem cells to clinically relevant doses of cisplatin and paclitaxel 64. It has previously been reported that mutation of RB1 is significantly related to patient survival and early HCC recurrence after resection, and is an independent predictor of early recurrence after liver cancer surgery^49^. The inactivation of RB1 leads to the activation of E2F1, causing tumor cell proliferation^50^. In this study, the inhibitors CDK2-IN-73 and Palbociclib could reverse the drug resistance effect caused by RB1 signaling, and Palbociclib could selectively inhibit CDK4/6. Approved to be marketed for the treatment of breast and gastric cancer, we found that Palbociclib could reverse the resistance of apatinib to HCC, providing a new option for the treatment of HCC patients with resistance to apatinib.

Through the comprehensive analysis of the epigenome, transcriptome, and proteome of drug-resistant and wild-type cells, and verification at the cellular level and in clinical samples, we found that the mechanism of apatinib resistance in HCC depends on changes in RB1 signaling. The inhibitors CDK2-IN-73 and Palbociclib could reverse the drug resistance caused by RB1 signaling. This new perspective is expected to provide a solid theoretical basis for subsequent clinical trials in patients with liver cancer who are resistant to apatinib.

## Supplementary Material

Supplementary figures and tables.

## References

[1] Siegel RL, Miller KD, Jemal A (2019). Cancer statistics, 2019. CA Cancer J Clin.

[2] Wen S, Shao G, Zheng J, Zeng H, Luo J, Gu D (2019). Apatinib regulates the cell proliferation and apoptosis of liver cancer by regulation of VEGFR2/STAT3 signaling. Pathol Res Pract.

[3] Wang Y, He L, Du Y, Zhu P, Huang G, Luo J (2015). The long noncoding RNA lncTCF7 promotes self-renewal of human liver cancer stem cells through activation of Wnt signaling. Cell Stem Cell.

[4] Ma KW, Chan A, Chok K, Tan TC, Lo CM (2019). Liver Transplantation - The Best and Last Chance of Cure for Hepatocellular Carcinoma with Portal Vein Invasion. HPB.

[5] Zhang L, Chen L, Yu H (2019). Phase II study of apatinib, a novel tyrosine kinase inhibitor targeting tumor angiogenesis, as second-line treatment for recurrent or advanced cervical cancer patients. Invest New Drugs.

[6] Xue P, Wang N, Mao Y, Zhu S (2019). Anti-angiogenesis treatment in a patient with appendix metastasis of small cell lung cancer: A case report. Medicine (Baltimore).

[7] Teleanu RI, Chircov C, Grumezescu AM, Teleanu DM (2019). Tumor Angiogenesis and Anti-Angiogenic Strategies for Cancer Treatment. J Clin Med.

[8] Kou P, Zhang Y, Shao W, Zhu H, Zhang J, Wang H (2017). Significant efficacy and well safety of apatinib in an advanced liver cancer patient: a case report and literature review. Oncotarget.

[9] Li J, Qin S, Xu J, Xiong J, Wu C, Bai Y (2016). Randomized, Double-Blind, Placebo-Controlled Phase III Trial of Apatinib in Patients with Chemotherapy-Refractory Advanced or Metastatic Adenocarcinoma of the Stomach or Gastroesophageal Junction. J Clin Oncol.

[10] Qin S (2014). Apatinib in Chinese patients with advanced hepatocellular carcinoma: A phase II randomized, open-label trial. Journal of Clinical Oncology.

[11] Cabrera C, Clotet B (2005). Mutations resulting in resistence of antiretroviral drugs in HIV-infected patients. Med Clin-Barcelona.

[12] Gomes RA, Ramirez RRA, Maciel JKD, Agra MD, de Souza MDV, Falcao-Silva VS (2011). PHENOLIC COMPOUNDS FROM Sidastrum micranthum (A. St.-Hil.) FRYXELL AND EVALUATION OF ACACETIN AND 7,4 '-DI-O-METHYLISOSCUTELLAREIN AS MOTULATOR OF BACTERIAL DRUG RESISTENCE. Quim Nova.

[13] Stuyver L, Van Geyt C, De Gendt S, Van Reybroeck G, Zoulim F, Leroux-Roels G (1999). A line probe assay for monitoring drug-resistence in hepatitis B virus (HBV) infected patients during antiviral therapy. Hepatology.

[14] Zelnickova J, Nejdl L, Richtera L, Kopel P, Adam V (2018). Interaction of Platinum-Based Cytostatics and Platinum Nanoparticles with Metallothionein - Potencial Source of the Antitumor Drug Resistence. 9th International Conference on Nanomaterials - Research & Application (Nanocon 2017).

[15] Zhou C, Kong W, Ju T, Xie Q, Zhai L (2020). MiR-185-3p mimic promotes the chemosensitivity of CRC cells via AQP5. Cancer Biol Ther.

[16] Wang B, Shen C, Li Y, Zhang T, Huang H, Ren J (2019). Oridonin overcomes the gemcitabine resistant PANC-1/Gem cells by regulating GST pi and LRP/1 ERK/JNK signalling. Onco Targets Ther.

[17] Zhou H, Dai Z, Li J, Wang J, Zhu H, Chang X (2023). TMBIM6 prevents VDAC1 multimerization and improves mitochondrial quality control to reduce sepsis-related myocardial injury. Metabolism.

[18] Zou R, Tao J, Qiu J, Lu H, Wu J, Zhu H (2022). DNA-PKcs promotes sepsis-induced multiple organ failure by triggering mitochondrial dysfunction. Journal of Advanced Research.

[19] Zou R, Shi W, Qiu J, Zhou N, Du N, Zhou H (2022). Empagliflozin attenuates cardiac microvascular ischemia/reperfusion injury through improving mitochondrial homeostasis. Cardiovasc Diabetol.

[20] Wang S, Zhu H, Li R, Mui D, Toan S, Chang X (2022). DNA-PKcs interacts with and phosphorylates Fis1 to induce mitochondrial fragmentation in tubular cells during acute kidney injury. Sci Signal.

[21] Ma L, Zou R, Shi W, Zhou N, Chen S, Zhou H (2022). SGLT2 inhibitor dapagliflozin reduces endothelial dysfunction and microvascular damage during cardiac ischemia/reperfusion injury through normalizing the XO-SERCA2-CaMKII-coffilin pathways. Theranostics.

[22] Zhu H, Tan Y, Du W, Li Y, Toan S, Mui D (2021). Phosphoglycerate mutase 5 exacerbates cardiac ischemia-reperfusion injury through disrupting mitochondrial quality control. Redox Biol.

[23] Langdon WB (2015). Performance of genetic programming optimised Bowtie2 on genome comparison and analytic testing (GCAT) benchmarks. BioData Min.

[24] Zhang Y, Liu T, Meyer CA, Eeckhoute J, Johnson DS, Bernstein BE (2008). Model-based analysis of ChIP-Seq (MACS). Genome Biol.

[25] Kim D, Langmead B, Salzberg SL (2015). HISAT: a fast spliced aligner with low memory requirements. Nat Methods.

[26] Huang da W, Sherman BT, Lempicki RA (2009). Systematic and integrative analysis of large gene lists using DAVID bioinformatics resources. Nat Protoc.

[27] Thorvaldsdottir H, Robinson JT, Mesirov JP (2013). Integrative Genomics Viewer (IGV): high-performance genomics data visualization and exploration. Brief Bioinform.

[28] Love MI, Huber W, Anders S (2014). Moderated estimation of fold change and dispersion for RNA-seq data with DESeq2. Genome Biol.

[29] Anders S, Pyl PT, Huber W (2015). HTSeq-a Python framework to work with high-throughput sequencing data. Bioinformatics.

[30] Sinitcyn P, Tiwary S, Rudolph J, Gutenbrunner P, Wichmann C, Yilmaz S (2018). MaxQuant goes Linux. Nat Methods.

[31] Zhou T, Li C, Zhao W, Wang X, Wang F, Sha J (2016). MaxReport: An Enhanced Proteomic Result Reporting Tool for MaxQuant. PLoS One.

[32] Boillot F, Chabin A, Bure C, Venet M, Belsky A, Bertrand-Urbaniak M (2002). The Perseus Exobiology mission on MIR: behaviour of amino acids and peptides in Earth orbit. Orig Life Evol Biosph.

[33] Szklarczyk D, Morris JH, Cook H, Kuhn M, Wyder S, Simonovic M (2017). The STRING database in 2017: quality-controlled protein-protein association networks, made broadly accessible. Nucleic Acids Res.

[34] Zhou H, Toan S, Zhu P, Wang J, Ren J, Zhang Y (2020). DNA-PKcs promotes cardiac ischemia reperfusion injury through mitigating BI-1-governed mitochondrial homeostasis. Basic Res Cardiol.

[35] Wang J, Zhu P, Toan S, Li R, Ren J, Zhou H (2020). Pum2-Mff axis fine-tunes mitochondrial quality control in acute ischemic kidney injury. Cell Biol Toxicol.

[36] Wang J, Zhu P, Li R, Ren J, Zhou H (2020). Fundc1-dependent mitophagy is obligatory to ischemic preconditioning-conferred renoprotection in ischemic AKI via suppression of Drp1-mediated mitochondrial fission. Redox Biol.

[37] Wang J, Zhu P, Li R, Ren J, Zhang Y, Zhou H (2020). Bax inhibitor 1 preserves mitochondrial homeostasis in acute kidney injury through promoting mitochondrial retention of PHB2. Theranostics.

[38] Harding HP, Zhang Y, Scheuner D, Chen JJ, Kaufman RJ, Ron D (2009). Ppp1r15 gene knockout reveals an essential role for translation initiation factor 2 alpha (eIF2alpha) dephosphorylation in mammalian development. Proc Natl Acad Sci U S A.

[39] Flaherty RL, Falcinelli M, Flint MS (2019). Stress and drug resistance in cancer. Cancer Drug Resistance.

[40] Cameron AC, Touyz RM, Lang NN (2016). Vascular Complications of Cancer Chemotherapy. Can J Cardiol.

[41] Liu J, Gefen O, Ronin I, Bar-Meir M, Balaban NQ (2020). Effect of tolerance on the evolution of antibiotic resistance under drug combinations. Science.

[42] Su XZ, Lane KD, Xia L, Sa JM, Wellems TE (2019). Plasmodium Genomics and Genetics: New Insights into Malaria Pathogenesis, Drug Resistance, Epidemiology, and Evolution. Clin Microbiol Rev.

[43] Shah KN, Bandyopadhyay S (2019). Targeting the evolution of drug resistance in lung cancer. Mol Cell Oncol.

[44] Kyrochristos ID, Ziogas DE, Roukos DH (2019). Drug resistance: origins, evolution and characterization of genomic clones and the tumor ecosystem to optimize precise individualized therapy. Drug Discov Today.

[45] Bakhiet AMA, Abdelraheem MH, Kheir A, Omer S, Gismelseed L, Abdel-Muhsin AA (2019). Evolution of Plasmodium falciparum drug resistance genes following artemisinin combination therapy in Sudan. Trans R Soc Trop Med Hyg.

[46] Nadiradze G, Horvath P, Sautkin Y, Archid R, Weinreich FJ, Konigsrainer A (2019). Overcoming Drug Resistance by Taking Advantage of Physical Principles: Pressurized Intraperitoneal Aerosol Chemotherapy (PIPAC). Cancers (Basel).

[47] Makovec T (2019). Cisplatin and beyond: molecular mechanisms of action and drug resistance development in cancer chemotherapy. Radiol Oncol.

[48] Chen SZ, Lin KN, Xiao M, Luo XF, Li Q, Ren JH (2017). [Distribution and drug resistance of pathogens of blood stream infection in patients with hematological malignancies after chemotherapy]. Zhonghua Xue Ye Xue Za Zhi.

[49] Echeverria GV, Ge Z, Seth S, Zhang X, Jeter-Jones S, Zhou X (2019). Resistance to neoadjuvant chemotherapy in triple-negative breast cancer mediated by a reversible drug-tolerant state. Sci Transl Med.

[50] Yuan R, Hou Y, Sun W, Yu J, Liu X, Niu Y (2017). Natural products to prevent drug resistance in cancer chemotherapy: a review. Ann N Y Acad Sci.

[51] Leontiou C, Lakey JH, Lightowlers R, Turnbull RM, Austin CA (2006). Mutation P732L in human DNA topoisomerase IIbeta abolishes DNA cleavage in the presence of calcium and confers drug resistance. Mol Pharmacol.

[52] Losasso C, Cretaio E, Fiorani P, D'Annessa I, Chillemi G, Benedetti P (2008). A single mutation in the 729 residue modulates human DNA topoisomerase IB DNA binding and drug resistance. Nucleic Acids Res.

[53] Beaumont KA, Hill DS, Daignault SM, Lui GYL, Sharp DM, Gabrielli B (2016). Cell Cycle Phase-Specific Drug Resistance as an Escape Mechanism of Melanoma Cells. J Invest Dermatol.

[54] Gamell C, Schofield AV, Suryadinata R, Sarcevic B, Bernard O (2013). LIMK2 mediates resistance to chemotherapeutic drugs in neuroblastoma cells through regulation of drug-induced cell cycle arrest. PLoS One.

[55] Li J, Kolberg K, Schlecht U, St Onge RP, Aparicio AM, Horecka J (2019). A biosensor-based approach reveals links between efflux pump expression and cell cycle regulation in pleiotropic drug resistance of yeast. J Biol Chem.

[56] Luo L, Gao W, Wang J, Wang D, Peng X, Jia Z (2018). Study on the Mechanism of Cell Cycle Checkpoint Kinase 2 (CHEK2) Gene Dysfunction in Chemotherapeutic Drug Resistance of Triple Negative Breast Cancer Cells. Med Sci Monit.

[57] Xie JL, Qin L, Miao Z, Grys BT, Diaz JC, Ting K (2017). The Candida albicans transcription factor Cas5 couples stress responses, drug resistance and cell cycle regulation. Nat Commun.

[58] Brummer C, Faerber S, Bruss C, Blank C, Lacroix R, Haferkamp S (2019). Metabolic targeting synergizes with MAPK inhibition and delays drug resistance in melanoma. Cancer Lett.

[59] Guggisberg AM, Frasse PM, Jezewski AJ, Kafai NM, Gandhi AY, Erlinger SJ (2018). Suppression of Drug Resistance Reveals a Genetic Mechanism of Metabolic Plasticity in Malaria Parasites. mBio.

[60] Pan Y, Cao M, Liu J, Yang Q, Miao X, Go VLW (2017). Metabolic Regulation in Mitochondria and Drug Resistance. Adv Exp Med Biol.

[61] Tan W, Zhong Z, Carney RP, Men Y, Li J, Pan T (2019). Deciphering the metabolic role of AMPK in cancer multi-drug resistance. Semin Cancer Biol.

[62] Le F, Luo P, Yang QO, Zhong XM (2017). MiR-181a promotes growth of thyroid cancer cells by targeting tumor suppressor RB1. Eur Rev Med Pharmacol Sci.

[63] Sourvinos G, Kazanis I, Delakas D, Cranidis A, Spandidos DA (2001). Genetic detection of bladder cancer by microsatellite analysis of p16, RB1 and p53 tumor suppressor genes. J Urol.

[64] Deng S, Wong CKC, Lai HC, Wong AST (2017). Ginsenoside-Rb1 targets chemotherapy-resistant ovarian cancer stem cells via simultaneous inhibition of Wnt/beta-catenin signaling and epithelial-to-mesenchymal transition. Oncotarget.

