# Supplementary material for: Integrative analysis of multi-omics data reveals inhibition of RB1 signaling promotes apatinib resistance of hepatocellular carcinoma
Source: Int J Biol Sci. 2023 Aug 21;19(14):4511–24. doi: 10.7150/ijbs.83862 (PMC10535702; doi:10.7150/ijbs.83862)
Supplement: Supplementary file 1 — Supplementary figures and tables. [file ijbsv19p4511s1.zip › Supplemental Figures and Tables/Supplemental Figures.pdf]

Supplemental Figure 1

Target Sequences	Name	P-value	log P-pvalue	q-value (Benjamini)
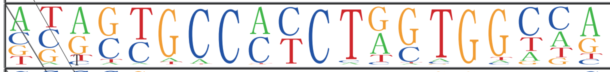	CTCF(Zf)/CD4+-CTCF-ChIP-Seq(Barski_et_al.)/Homer	1e-134	-3.108e+02	0.0000
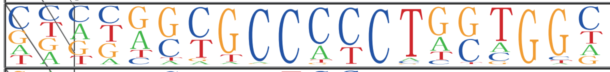	BORIS(Zf)/K562-CTCFL-ChIP-Seq(GSE32465)/Homer	1e-109	-2.517e+02	0.0000
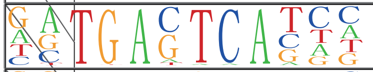	Jun-AP1(bZIP)/K562-cJun-ChIP-Seq(GSE31477)/Homer	1e-50	-1.162e+02	0.0000
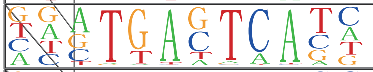	Fra1(bZIP)/BT549-Fra1-ChIP-Seq(GSE46166)/Homer	1e-49	-1.133e+02	0.0000
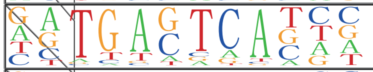	Fosl2(bZIP)/3T3L1-Fosl2-ChIP-Seq(GSE56872)/Homer	1e-48	-1.113e+02	0.0000
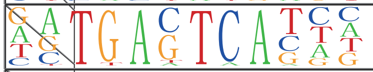	Atf3(bZIP)/GBM-ATF3-ChIP-Seq(GSE33912)/Homer	1e-46	-1.067e+02	0.0000
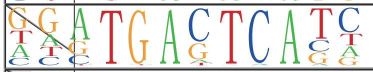	Fra2(bZIP)/Striatum-Fra2-ChIP-Seq(GSE43429)/Homer	1e-46	-1.060e+02	0.0000
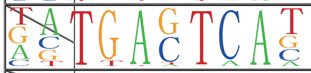	BATF(bZIP)/Th17-BATF-ChIP-Seq(GSE39756)/Homer	1e-43	-9.918e+01	0.0000
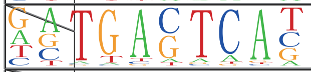	JunB(bZIP)/DendriticCells-Junb-ChIP-Seq(GSE36099)/Homer	1e-39	-9.106e+01	0.0000
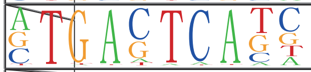	AP-1(bZIP)/ThioMac-PU.1-ChIP-Seq(GSE21512)/Homer	1e-34	-7.983e+01	0.0000
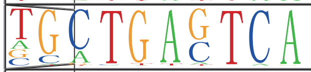	Bach2(bZIP)/OCILy7-Bach2-ChIP-Seq(GSE44420)/Homer	1e-31	-7.326e+01	0.0000
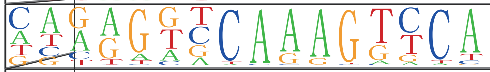	HNF4a(NR),DR1/HepG2-HNF4a-ChIP-Seq(GSE25021)/Homer	1e-28	-6.560e+01	0.0000
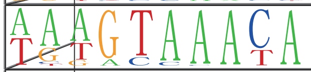	FOXA1(Forkhead)/MCF7-FOXA1-ChIP-Seq(GSE26831)/Homer	1e-27	-6.296e+01	0.0000
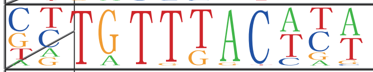	Foxa2(Forkhead)/Liver-Foxa2-ChIP-Seq(GSE25694)/Homer	1e-26	-6.197e+01	0.0000
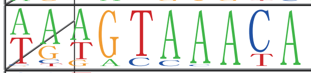	FOXA1(Forkhead)/LNCAP-FOXA1-ChIP-Seq(GSE27824)/Homer	1e-25	-5.922e+01	0.0000
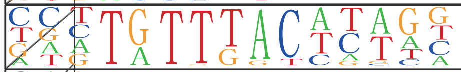	Foxa3(Forkhead)/Liver-Foxa3-ChIP-Seq(GSE77670)/Homer	1e-24	-5.714e+01	0.0000
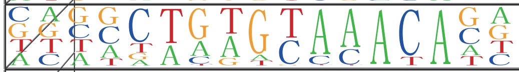	Fox:Ebox(Forkhead,bHLH)/Panc1-Foxa2-ChIP-Seq(GSE47459)/Homer	1e-23	-5.386e+01	0.0000
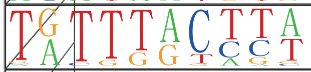	FOXM1(Forkhead)/MCF7-FOXM1-ChIP-Seq(GSE72977)/Homer	1e-23	-5.365e+01	0.0000
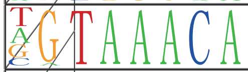	Foxo3(Forkhead)/U2OS-Foxo3-ChIP-Seq(E-MTAB-2701)/Homer	1e-16	-3.714e+01	0.0000
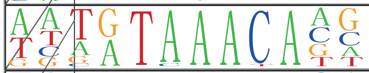	FoxL2(Forkhead)/Ovary-FoxL2-ChIP-Seq(GSE60858)/Homer	1e-14	-3.266e+01	0.0000
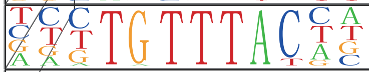	FOXP1(Forkhead)/H9-FOXP1-ChIP-Seq(GSE31006)/Homer	1e-14	-3.251e+01	0.0000
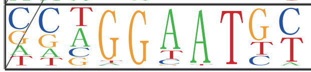	TEAD4(TEA)/Tropoblast-Tead4-ChIP-Seq(GSE37350)/Homer	1e-12	-2.951e+01	0.0000

19

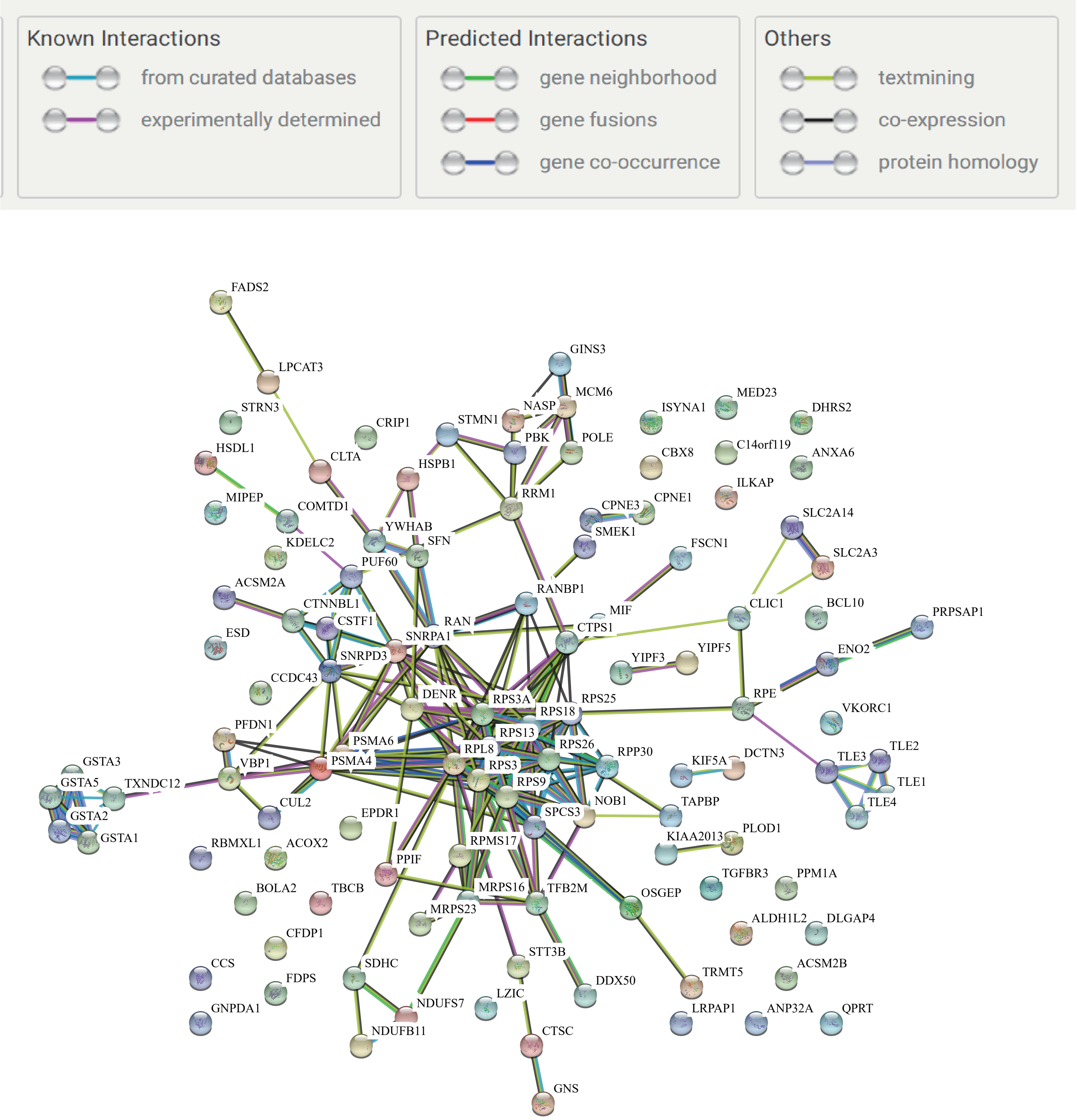

Supplemental Figure 2
